# Evaluation of Two Alloplastic Biomaterials in a Critical-Size Rat Calvarial Defect Model

**DOI:** 10.3390/jfb16060214

**Published:** 2025-06-06

**Authors:** Amanda Finger Stadler, Marta Liliana Musskopf, Vishal Gohel, Jonathan Reside, Eric Everett, Patricia Miguez, Cristiano Susin

**Affiliations:** 1 Department of Periodontology, Endodontics and Dental Hygiene, Adams School of Dentistry, University of North Carolina at Chapel Hill, Chapel Hill, NC 27599, USA; 2 Department of Biomedical Sciences, Adams School of Dentistry, University of North Carolina at Chapel Hill, Chapel Hill, NC 27517, USA

**Keywords:** bone biomaterials, critical-size defect, rat calvaria, tissue engineering

## Abstract

Aim: to evaluate the bone regeneration capacity of two alloplastic biomaterials in a critical-size rat calvarial defect model. Methods: A total of 80 rats were randomized into 8 groups of 10 animals each. An Ø8 mm, critical-size calvarial defect was created, and the following treatments were randomly allocated: sham surgery, deproteinized bovine bone mineral (DBBM) + collagen membrane (CM), poly-(lactic-co-glycolic-acid) (PLGA)-coated pure phase β-tricalcium phosphate (β-TCP), or PLGA-coated 60% hydroxyapatite (HA):40%β-TCP. Animals were allowed to heal for 2 and 6 weeks. Microcomputed tomography (μCT) was used to evaluate mineralized tissue and biomaterial displacement. Histological samples were used to evaluate new bone formation. Results: μCT analysis showed no significant differences among groups for total volume of mineralized tissue or residual biomaterials. DBBM + CM showed significantly increased horizontal biomaterial displacement at 2 weeks but not at 6 weeks. Histological analysis showed that sham surgery had a significantly higher percentage of bone area fraction than the DBBM + CM and PLGA + β-TCP at 2 weeks, but not at 6 weeks. Residual biomaterial area fraction showed no significant differences among experimental groups at any healing time. Conclusions: The alloplastic biomaterials showed suitable construct integrity and retention in the defect. All biomaterials were associated with limited new bone formation comparable to the sham surgery control.

## 1. Introduction

The dimensional changes on the hard and soft tissues that occur secondary to tooth loss, periodontal disease, trauma, and oral cancer often preclude the ideal prosthetic oral rehabilitation. Thus, bone augmentation is often necessary to reconstruct the alveolar ridge architecture [1]. Whereas autogenous bone is still considered the gold standard for alveolar bone regeneration [2,3], limited intra-oral sources and increased comorbidity limit its use in daily practice. In this context, different bone biomaterials and substitutes have been developed to replace or extend autogenous bone for alveolar ridge augmentation. Several bone biomaterials are commercially available and have been used in dentistry, including allogeneic, xenogeneic, and alloplastic biomaterials [4]. Osteogenic potential, dimensional stability, retention, patient acceptance, handling, and cost are important considerations for biomaterials selection.

Small and large animal models have been used to test the osteogenic potential of biologics and bone biomaterials. The critical-size rat calvarial defect model is a well-established model that allows for the radiographic and histological evaluation of bone regeneration in a challenging craniofacial bony defect [5]. Several growth factors [6,7,8] and bone biomaterials [9] have been tested using this model with mixed results.

Two synthetic bone biomaterials with improved handling and in situ hardening have been developed: poly-(lactic-co-glycolic-acid) (PLGA)-coated pure phase β-tricalcium phosphate (β-TCP, PLGA + β-TCP) and PLGA-coated 60% hydroxyapatite (HA) and 40% β-TCP (PLGA + HA/β-TCP). Pre-clinical studies using these biomaterials show biocompatibility and improved new bone formation when compared to empty controls [10,11,12], and case series suggest implant stability for up to 8 months in previously augmented sites [13,14]. The aim of this study was to compare these synthetic biomaterials with an industry-leading deproteinized bovine bone mineral (DBBM) covered by a collagen membrane (CM) using a critical-size rat calvarial defect model. New bone formation, construct integrity, and biomaterial displacement were evaluated. Our hypothesis was that the new biomaterials would yield comparable new bone formation but better geometry and less biomaterial displacement due to the in situ hardening property.

## 2. Materials and Methods

### 2.1. Animals

Eighty male Sprague Dawley rats (Rattus norvegicus), age 11–12 weeks, weight 310–510 g (mean 369 g), obtained from Charles River Labs (Wilmington, MA, USA), were used. Housing, husbandry, and experimental manipulations were performed following the National Research Council’s Guide for Care and Use of Laboratory Animals and a protocol approved for this study by the Institutional Animal Care and Use Committee, University of North Carolina at Chapel Hill.

The animals were acclimatized for at least 7 days and single-housed in individual, autoclaved cages. The cages were kept in a temperature-controlled setting with a twelve-hour light/dark cycle, monitored daily. The animals had ad libitum access to water and a standard laboratory diet. This report was prepared in harmony with ARRIVE guidelines for reporting animal research [15].

### 2.2. Biomaterials

This study compared two synthetic biomaterials and a reference biomaterial: (a) PLGA + β-TCP (Easy-Graft Classic, Degradable Solutions AG, Schlieren, Switzerland); (b) PLGA + HA/β-TCP (Easy-Graft Crystal, Degradable Solutions AG, Schlieren, Switzerland); (c) DBBM (Bio-Oss, Geistlich, Wolhusen, Switzerland). The DBBM was used in combination with a non-chemically cross-linked, resorbable, porcine collagen barrier membrane (CM, Bio-Gide, Geistlich, Wolhusen, Switzerland) following clinical practice (Appendix A, Table A1).

### 2.3. Study Design and Sample

Animals were randomly assigned into 8 groups of 10 animals each (four treatment groups and two healing times). Animals received one of the following treatments: (a) PLGA + β-TCP; (b) PLGA + HA/β-TCP; (c) DBBM + CM; and (d) sham surgery. Animals were allowed to heal for 2 or 6 weeks. A surgical assistant not involved with the surgeries prepared the biomaterials using a pre-formed template of Ø8 mm × 1 mm height to ensure the volume of biomaterial was consistent for every group; the surgical assistant randomly assigned the biomaterials to the animals. The surgeons adhered to this specific protocol to the extent possible given the visual characteristics of the biomaterials. Based on experience from previous studies [6,9,16], it was estimated that a sample size of 10 animals per group was necessary to achieve 80% power to detect a mean difference of 30% in defect closure between experimental groups. A two-sided two-sample *t*-test with a significance level of 5% was used for the sample size calculation (Stata 17.1 for Mac, Stata Corporation, College Station, TX, USA).

### 2.4. Surgical Procedures

The animals were weighed and pre-medicated with subcutaneous buprenorphine (0.01–0.05 mg/kg) to facilitate postoperative analgesia and intramuscular cefazolin (15–25 mg/kg) for antibiotic prophylaxis. Anesthesia was induced using injectable ketamine (6–75 mg/kg) and dexmedetomidine (0.5–0.75 mg/kg) by experienced veterinary technicians from the UNC Animal Studies Core Facility. The appropriate level of anesthesia was confirmed by the toe pinch reflex test. An ear tag identification was placed. The skull of the animals was shaved and disinfected using an iodine solution and alcohol pad, and the eyes of the animals were protected with the application of ophthalmic ointment. Animals were moved to the surgical area and stabilized using a stereotaxic table (Stoelting, Wood Dale, IL, USA). The animals were surgically draped, and a heating pad was positioned below the animal to provide warmth during the procedure. Trans-surgical anesthesia depth was assessed using a heart rate and oxygen saturation monitor, as well as physical signs of breathing and toe pinch reflex.

Four experienced surgeons (C.S., A.F.S., M.L.M., and V.G.) performed all surgeries with aseptic techniques and conditions. Detailed surgical procedures, including photographs of surgical procedures, are described elsewhere [6]. Briefly, a 15–20 mm midline incision was performed through the skin along the sagittal suture of the skull, followed by detachment of the soft tissues and periosteum. Under sterile water irrigation, a critical-size, Ø8 mm, through–through, osteotomy defect centered over the sagittal suture between lambda and bregma was created using a Ø8-mm diamond-coated trephine bur (Continental Diamond Tool Corporation, New Haven, CT, USA). Care was taken to keep the dura mater and sagittal sinus intact [5]. Excessive bleeding was controlled with moist gauze pressure, absorbable collagen sponge, and silver nitrate. The defects were then filled with one of the test materials, DBBM (reference), or left empty to fill naturally with a blood clot (sham surgery). In the DBBM group, a trimmed collagen barrier membrane was placed covering the biomaterial and overlapped the edges of the defect by approximately 1 mm. The full-thickness flaps were approximated, and closure with everted wound margins was obtained using surgical staples (Weck Visistat Disposable Skin Staples, Teleflex, Morrisville, NC, USA) for primary intention healing.

### 2.5. Postsurgery Procedures

The animals were given an intraperitoneal injection of Atipamezole (0.5–1.5 mg/kg) to reverse the effects of anesthesia and placed in empty cages equipped with heating pads to monitor recovery. Carprofen (5 mg/kg) was administered at 24 and 48 h after surgery for pain control. Postsurgical antibiotics were given once daily for 3 days (intramuscular cefazolin 15–25 mg/kg). Animals that exhibited signs of pain beyond day 3 were given additional doses as determined by the attending veterinarian. All animals were monitored twice daily for the first 72 h post-surgery by trained personnel for any signs of infection, pain, or surgical complication. Daily monitoring of each animal was performed thereafter.

Removal of surgical staples was performed under anesthesia with isoflurane in an induction chamber 7–10 days after surgery. The animals were euthanized either at 2 or 6 weeks of healing time using a CO_2_ induction chamber followed by thoracotomy as a secondary method. The calvaria bone was carefully harvested after soft tissue dissection and fixated for 3 days under agitation in a 10% buffered formalin solution before radiographic analysis.

### 2.6. Radiographic Evaluation

Calvarial specimens were scanned using an ex vivo desktop X-ray microcomputed tomography system (μCT, SkyScan 1275, Bruker, Kontich, Belgium), equipped with an x-ray tube with 100 Kv potential and a camera pixel size of 75 um. Each sample was positioned in a cylinder sample holder with the sagittal suture oriented perpendicular to the image position. Scanning was acquired with samples wrapped in gauze and immersed in phosphate-buffered saline (PBS) solution, using 70 Kv, 142 uA energy, 16 μm magnification, an Alumina filter of 1 mm, 52 ms integration time, a 0.5 rotation step degree, 360-degree rotation, random movement, and 5 frame averaging. Three-dimensional reconstructions were performed on NRecon software (Version 1.7.4.6, Bruker Micro-CT, Cambridge, UK), using 20% beam hardening and the same dynamic range. Alignment of acquired images was performed with DataViewer software (Version 1.5.6.2, Bruker Micro-CT, Cambridge, UK) [6,17].

#### 2.6.1. Mineralized Tissue Volume/Total Volume and Biomaterial Volume/Total Volume

Using CTAnalyzer software (Version 1.18.8.0, Bruker Micro-CT, Cambridge, UK), a cylindrical region of interest with a diameter of Ø7.5 mm and height of 1 mm was selected for analysis (63 slices) using a coronal view. A threshold of 80–255 was used for segmentation of the total mineralized tissue volume. Thresholds of 140–240, 140–255, and 150–255 were used for segmentation of biomaterials DBBM + CM, PLGA + β-TCP, and PLGA + HA/β-TCP, respectively.

#### 2.6.2. Biomaterial Displacement

Displacement of biomaterial was measured on CTAn software using the coronal sections. For measuring horizontal displacement, a volume of interest of Ø10 and Ø12 mm × 1 mm height was created at the top of the defect. The Ø10 × 1 mm height volume was then subtracted from the Ø12 × 1 mm height, generating a horizontal displacement measurement area of 2 mm around the defect. For the vertical displacement, a volume of interest of Ø7.5 × 1 mm height was created 1 mm above the defect. The same thresholds used for segmentation of biomaterials within the defect were used for measurement of biomaterial displacement.

### 2.7. Histological Preparation

Calvaria biopsies were processed for demineralized histology and incandescent and polarized light microscopy analysis. Samples were demineralized in formic acid bone decalcifier 10% (10% Immunocal Decalcifier, StatLab Medical Products, McKinney, TX, USA, 90% distilled water) for 14–28 days at room temperature with a new solution every 3 days. Samples were washed, dehydrated in a graded series of ethyl alcohol (70–100%), cleared in xylene, and embedded in paraffin perpendicular to the sagittal suture. Using a microtome, coronal sections of 6 µm thickness were obtained and mounted on Frost Plus glass slides. Standard protocols were used for Masson’s Trichrome staining.

### 2.8. Histomorphometric Analysis

Two experienced examiners (A.F.S. and C.S.) identified the landmarks and extent of bone formation using polarized and incandescent light microscopy (BX63, Olympus American, Melville, NY, USA). Two calibrated, experienced examiners (A.F.S. and M.L.M.) then performed the histometric analysis using the same microscope setting, equipped with a microscope digital camera system (DP74, Olympus America, Melville, NY, USA) and a PC-based image analysis software (cellSens Dimension 2.1 Digital Imaging Software, Olympus America, Melville, NY, USA). The following parameters were recorded for each defect site:

- Defect width: distance between the defect margins;
- Defect closure: fraction (%) of accumulated length of new bone formation between the defect margins;
- Defect area: area of regeneration including new bone formation, residual biomaterial and other tissue limited by the defect margins;
- Defect fill: total area of newly formed bone between the defect margins;
- Bone area fraction: fraction (%) of newly formed bone within the defect area;
- Residual biomaterial: total area of residual biomaterial between the defect margins;
- Biomaterial area fraction: fraction (%) of residual biomaterial within the defect area.

Examiners did not have access to group coding during the histological analysis; however, masking was not effective for all groups due to some biomaterials featuring unique characteristics.

### 2.9. Statistical Analysis

Statistical analysis was performed using statistical software (Stata 17.1 for Mac, Stata Corporation, College Station, TX, USA). Measures of central tendency (means and medians) and variability (standard deviations, 95% confidence intervals, percentiles, and range) were calculated and presented in tables. For the radiographic analysis, the primary outcome assessed was percentage of mineralized tissue volume (MTV) and biomaterial volume (BIO). The secondary experimental outcome assessed was horizontal and vertical biomaterial displacement measured in mm^3^. For the histological analysis, the primary outcome was linear defect closure, and the secondary outcomes were bone area fraction and biomaterial area fraction. Dunn’s test followed by Bonferroni’s correction was used to estimate *p*-values for comparison between groups. The level of significance was set at 5%.

Examiner reliability for the histomorphometric evaluation was assessed using the Intraclass Correlation Coefficient (ICC). This coefficient ranges between 0 and 1, and values close to 1 mean high reliability. The ICC was >0.94 for all parameters measured for both examiners.

## 3. Results

### 3.1. Surgical Outcomes

Fourteen animals succumbed during the surgery due to complications associated with anesthesia, and these animals were replaced. One animal died two days post-surgery and was not replaced (PLGA + β-TCP group at 2 weeks). The final sample size was 79 animals. Intraoperatively, 21 animals experienced bleeding that required the use of absorbable collagen sponge or silver nitrate to control bleeding. Five animals presented with post-operative edema. All other animals underwent surgical procedures uneventfully.

### 3.2. Radiographic Evaluation

**Figure 1:**
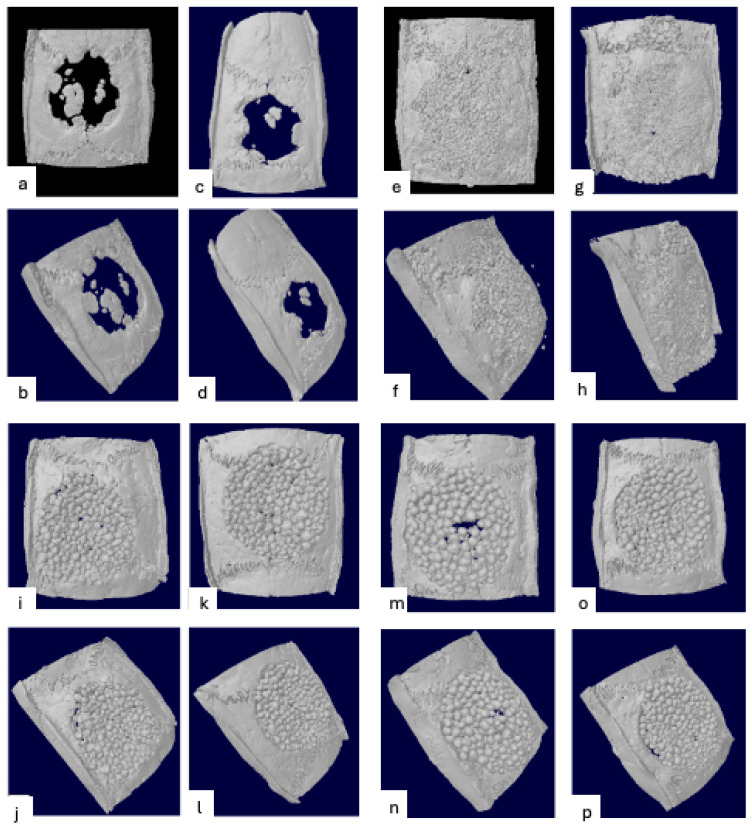
Representative microCT images of defect sites from each group at 2- and 6-week healing times: (**a**,**b**) sham surgery at 2 weeks; (**c**,**d**) sham surgery at 6 weeks; (**e**,**f**) DBBM + CM at 2 weeks; (**g**,**h**) DBBM + CM at 6 weeks; (**i**,**j**) PLGA + β-TCP at 2 weeks; (**k**,**l**) PLGA + β-TCP at 6 weeks; (**m**,**n**) PLGA + HA/β-TCP at 2 weeks; (**o**,**p**) PLGA + HA/β-TCP at 6 weeks.

**Figure 2:**
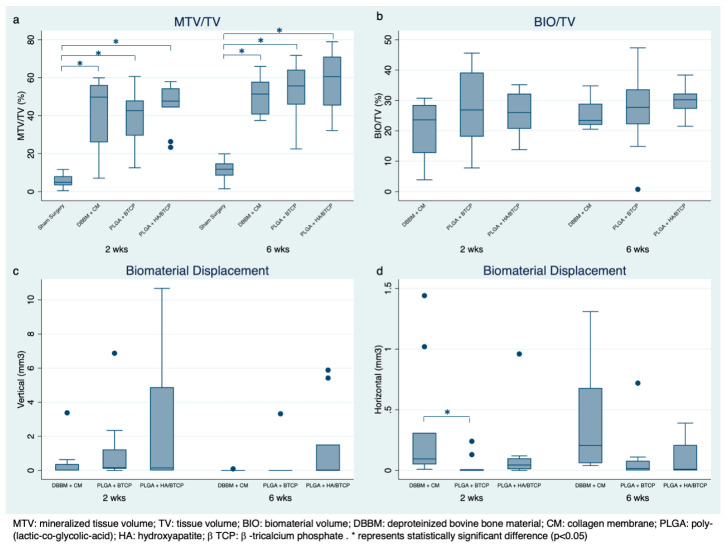
Box Plot from μCT analysis, according to experimental groups and healing time. (**a**) Percentage of mineralized tissue volume/tissue volume. (**b**) Percentage of biomaterial volume/tissue volume. (**c**) Volume of horizontal biomaterial displacement. (**d**) Volume of vertical biomaterial displacement. * represents statistically significant difference (*p* < 0.05).

Figure 1 depicts representative microCT images for each group at 2- and 6-week healing times. Figure 2a,b show the boxplot for total mineralized tissue and biomaterial volume according to experimental groups and healing times. The control group had significantly less mineralized tissue volume than the test groups at 2 weeks (5.48%) and 6 weeks (10.98%). The amount of mineralized tissue volume in the test groups ranged from 39.76% to 45.49% at 2 weeks and from 50.81% to 58.75% at 6 weeks. No significant differences were observed among the test groups. The mean biomaterial volume ranged from 21.43% to 27.18% at 2 weeks and between 25.24% and 30.40% at 6 weeks. No significant differences were found among test groups for either healing time. A significant increase in total mineralized tissue volume occurred between 2 and 6 weeks, but no significant differences were observed in the biomaterial volume between healing periods.

#### Biomaterial Displacement

**Figure A1:**
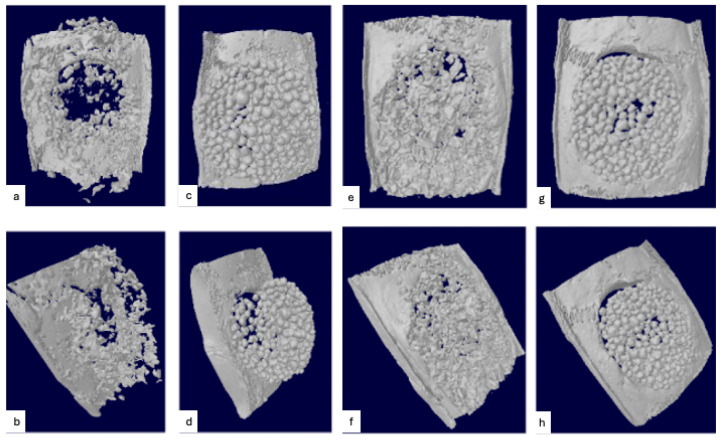
Representative microCT images of biomaterial displacement. (**a**,**b**) DBBM vertical displacement, (**c**,**d**) PLGA vertical displacement, (**e**,**f**) DBBM horizontal displacement, (**g**,**h**) PLGA horizontal displacement.

Figure 2c,d show the boxplot for vertical and horizontal displacement of the biomaterials according to experimental groups and healing time. No significant differences in vertical displacement were observed among groups at 2 and 6 weeks; the group PLGA + HA/β-TCP had the highest vertical displacement. At 6 weeks, vertical displacement was markedly reduced when compared to the 2 weeks. For horizontal biomaterial displacement, the DBBM + CM group had significantly higher displacement than the PLGA + β-TCP at 2 and 6 weeks (*p* = 0.009 and 0.024, respectively); no significant differences were observed between PLGA + HA/β-TCP and the other groups. Appendix B, Figure A1 shows a representative image of biomaterial displacement.

### 3.3. Histopathologic Observations

**Figure 3:**
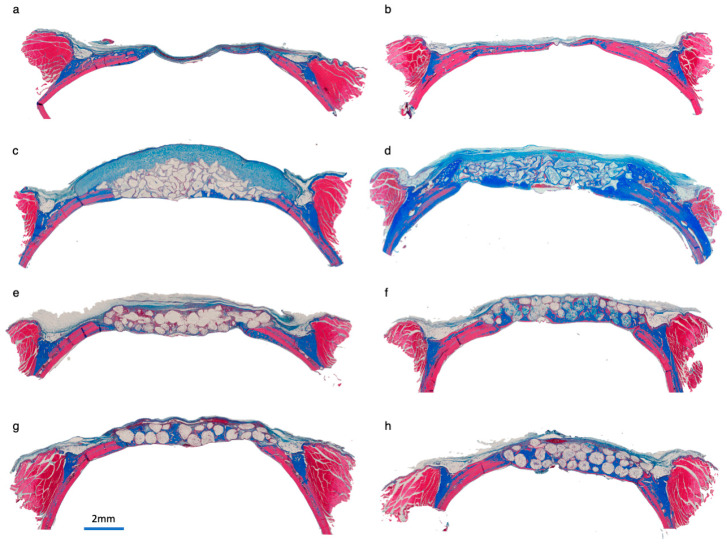
Masson’s Trichrome staining of new bone formation at defect sites from each group at 2- and 6-week healing times: (**a**) sham surgery at 2 weeks; (**b**) sham surgery at 6 weeks; (**c**) DBBM + CM at 2 weeks; (**d**) DBBM + CM at 6 weeks; (**e**) PLGA + β-TCP at 2 weeks; (**f**) PLGA + β-TCP at 6 weeks; (**g**) PLGA + HA/β-TCP at 2 weeks; (**h**) PLGA + HA/β-TCP at 6 weeks.

Figure 3 depicts Masson’s Trichrome staining of representative specimens for each group at 2- and 6-week healing times. New bone formation was observed extending from the defect margins along the lamina dura, surrounded by fibrovascular tissue in all groups.

#### 3.3.1. Sham Surgery

Sham surgery specimens showed new bone formation extending from the defect margins, usually filling about half of the defect. Bone was rarely observed at the central part of the defect, with the lamina dura and periosteum being in close contact (Figure 3a,b). None of the defects presented with complete closure of the defect.

#### 3.3.2. DBBM + CM

Specimens with DBBM + CM had biomaterial particles in close contact with new bone. At the central portion of the defect, biomaterial particles were surrounded by fibrovascular tissue. The CM was seen covering the defect at 2 weeks, cells infiltrating the matrix. The CM thickness had significantly decreased after 6 weeks of healing. No signs of biomaterial resorption were observed (Figure 3c,d). The DBBM particles were observed beyond the defect margins in some samples. CM appeared to induce woven-like bone to form from the edges of the defect in some samples by 6 weeks.

#### 3.3.3. PLGA + β-TCP and PLGA + HA/β-TCP

A cohesive construct was frequently observed for the PLGA + β-TCP (Figure 3e,f) and PLGA + HA/β-TCP (Figure 3g,h) groups. New bone was observed infiltrating the construct and in close contact with the particles, presenting as woven bone at 2 weeks and more lamellar bone at 6 weeks.

### 3.4. Histomorphometric Analysis

**Figure A2:**
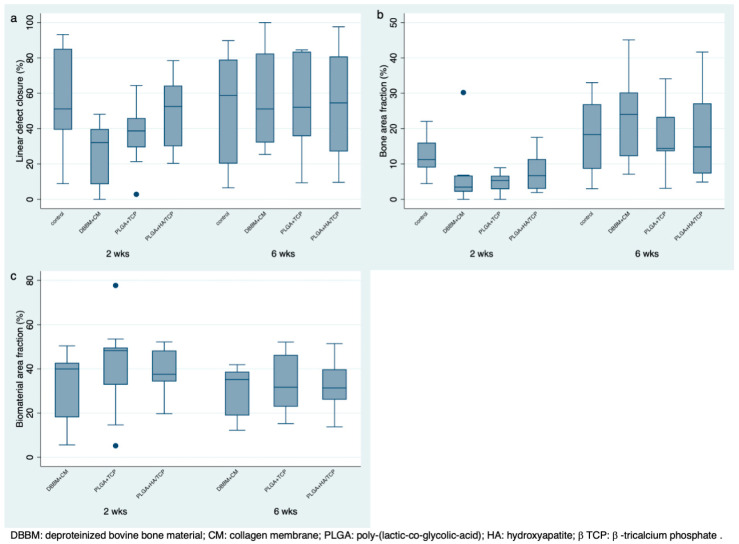
Box Plot from histological analysis, according to groups and healing period. (**a**) Percentage of linear defect bone closure. (**b**) Percentage of bone area fraction. (**c**) Percentage of biomaterial area fraction.

Table 1 summarizes the results for linear defect closure according to experimental group and healing time. At 2 weeks, the control group had significantly higher defect closure than the DBBM + CM group (*p* = 0.018); no other differences were observed. A high degree of variability was observed among the experimental groups. At 6 weeks, no statistically significant differences were observed among experimental groups, all groups having partial defect closure approximating 55%. Appendix B, Figure A2 shows a box plot depicting the percentage of linear defect bone closure.

Table 2 shows bone area fraction according to experimental group and healing time. At 2 weeks, the control group had statistically significantly higher percentage of bone area fraction than the DBBM + CM and PLGA + β-TCP groups (*p* = 0.007 and 0.010, respectively); no significant differences were observed between the control group and PLGA + HA/β-TCP. No significant differences among experimental groups were observed at 6 weeks. Appendix B, Figure A2 shows a box plot depicting the percentage of bone area fraction.

Table 3 presents biomaterial area fraction according to experimental group and healing time. The PLGA + β-TCP group presented with slightly increased residual biomaterial at 2- and 6-week healing times; however, no statistically significant differences were observed between groups at any healing time. Although the percentages of residual biomaterial presented with a tendency to decrease over time, no statistically significant differences were observed. Appendix B, Figure A2 shows a box plot depicting the percentage of biomaterial area fraction.

## 4. Discussion

This study compared two alloplastic bone biomaterials (PLGA + β-TCP and PLGA + HA/β-TCP) available for alveolar bone augmentation against a predicate biomaterial (DBBM + CM) using a critical-size rat calvaria defect model. Radiographic evaluation demonstrated that the bone biomaterials yielded a higher percentage of mineralized tissue volume at 2 weeks when compared to the control group, but no differences could be observed at 6 weeks. From a histological standpoint, the biomaterials demonstrated biocompatibility, represented by the close contact between biomaterial and bone, and the control group demonstrated enhanced linear bone closure and bone fraction area compared to the other experimental groups at 2 weeks; no significant differences were observed at 6 weeks. Bone biomaterial displacement was observed at 2 weeks (Appendix B, Figure A1 and Figure A2) but resolved after 6 weeks. In perspective, bone biomaterials did not enhance new bone formation beyond the innate regenerative potential of the defect.

The alloplastic biomaterials evaluated in this study (Table A1) are composed of HA and β-TCP, which are ceramics commonly used as bone substitutes for alveolar bone augmentation [18]. β-TCP presents good biocompatibility and low solubility and has been extensively used as a partially resorbable defect filler [18,19]. HA is a calcium phosphate biomaterial that has a composition similar to natural bone, is commonly used because of its osteoconductive properties, forms a chemical bond directly to the native bone once implanted, and has limited resorptive potential [20,21]. The β-TCP and the HA/β-TCP granules used in these materials are coated by PLGA, a biocompatible synthetic polymer that resorbs within 3–6 weeks and has no negative effects on bone formation [22]. Bizenjima et al., 2016 [10], in a critical size calvaria defect study, described proliferating cell nuclear antigen (PCNA)-positive cells around the biomaterials and penetrating the β-TCP granules at 4 weeks of healing, showing degradation of the PLGA outer layer. The authors also observed tartrate-resistant acid phosphatase staining (TRAP-positive osteoclast-like cells) at the surface of the biomaterials, suggesting remodeling activity of the biomaterial.

From a clinical perspective, the use of particulate bone biomaterials for alveolar ridge augmentation has become the treatment of choice for contained defects [23]. In non-contained defects, retention of particulate bone biomaterials at the surgical site and modeling of the reconstructed ridge can be very challenging. Non-resorbable and resorbable barriers and devices are usually used to address these issues, but their use increases surgical complexity, time, and costs [24]. In this study, β-TCP and HA + β-TCP coated with PLGA were used to improve the handling, geometry, and retention of the construct. Those materials contain a pre-filled syringe of polymer-coated granules and a separate ampule of polymer activator. The PLGA is plasticized by the activator and allows the biomaterial to agglutinate into a moldable construct that hardens in situ and creates a stable scaffold for tissue ingrowth and may potentially eliminate the need for a barrier membrane.

The displacement of bone biomaterials from the surgical site can affect wound stability and space provision, which are necessary for periodontal and alveolar bone healing and regeneration [25]. In this study, the PLGA + β-TCP group demonstrated significantly lower lateral displacement compared to the DBBM + CM group at 2 and 6 weeks. Moreover, PLGA + β-TCP and PLGA + HA/β-TCP biomaterials were able to maintain their cylindrical geometry for the 6-week healing period. Importantly, displacement of the whole construct instead of the granules was observed, which tended to resolve after 6 weeks.

A critical size defect is defined as a surgical defect that does not completely heal during the lifetime of the experimental animals [5]. Our laboratory has used the rat critical size calvarial defect to test biomaterials and biologics in support of bone formation [6,9,16]. The calvaria serves as a model for intramembranous bone formation in a cost-effective and timely manner [5,26]. Defect closure in sham surgery has ranged between 32.10% and 57.86% following up to 8 weeks of healing using this model [9,16], which is somewhat similar to the present findings. In comparison, allogeneic and synthetic bone biomaterials yielded a defect closure that ranged between 15.1% and 43.8% at 4 weeks [9]. On the other hand, rhBMP-2 alone or in combination with other growth factors can achieve complete defect closure depending on the dose and healing time [6,16].

An important limitation of this model is the standardization of the amount of biomaterial used in each defect. For this study, a template made of sterilized acetate was created, providing a space similar in size to the defect created on the calvaria in thickness and diameter. The amount of biomaterial dispensed was measured before the delivery to the defect. All materials were gently placed, and any excess material was removed to avoid displacement of the biomaterial and perforation of the dura. This pre-clinical model also limits the addition of a comparison gold standard group with autograft. The use of autograft as a comparison would be very challenging in a rat model due to the scarcity of bone that can be easily sourced with limited morbidity of the second surgical site.

The regenerative potential of the PLGA-coated β-TCP material has been previously studied in small and large animal models. Using a critical-size rat calvarial defect, Bizenjima et al. [10] showed significantly higher bone formation in the PLGA-coated β-TCP group when compared to the empty control at 6 weeks (59.9 ± 28.5% vs. 22.4 ± 10.4%). Similar amounts of residual β-TCP and PLGA + β-TCP were observed (~40%). Whereas a similar amount of residual biomaterial was observed in our study, no significant differences were observed for radiographic or histological bone formation. Using a rabbit calvarial critical size defect, Yip et al. [27] and Schmidlin et al. [11] did not find significant differences in bone formation between PLGA + HA/β-TCP and DBBM following 4 to 16 weeks of healing. Schmidlin et al. [11] reported similar results for PLGA + β-TCP. Wildburger et al. [22] compared particulate β-TCP and PLGA + HA/β-TCP using sinus floor augmentation in sheep and found a significantly higher new bone fraction for the PLGA + HA/β-TCP group. Naenni et al. [28] found no significant differences between PLGA + HA/β-TCP and biphasic calcium phosphate (60% HA and 40% β-TCP) on alveolar ridge augmentation using a dog model.

In a case series, Leventis et al. [14] evaluated core biopsies following the use of PLGA + β-TCP for alveolar ridge preservation after 4 months. They observed a mean bone volume of 24.36 ± 7.95% and residual bone biomaterial of 12.9 ± 7.7%. Whereas the bone volume observed herein is somewhat similar to the findings in humans (17.84 ± 9.39%), the residual biomaterial was substantially higher in our study (34.05 ± 13.15%). The discrepancy in residual bone biomaterial can be explained, at least in part, by the difference in healing time.

Reports from a randomized clinical trial comparing β-TCP coated with PLGA with a freeze-dried bone allograft for alveolar ridge preservation show that sites treated with β-TCP coated with PLGA presented with significantly less mineralized tissue; however, no site required further bone augmentation for dental implant placement, and there were no histologic or radiographic significant differences observed regarding the percent of remaining bone graft material and non-mineralized tissue content [29].

## 5. Conclusions

In conclusion, within the limitations of this study, all bone biomaterials were considered biocompatible and yielded comparable defect closure; however, none enhanced new bone formation beyond the innate regenerative potential of the defect. PLGA-coated β-TCP and HA/β-TCP provided additional benefits from a biomaterial handling, retention, and modeling standpoint.

## Figures and Tables

**Table 1:** Histological linear defect closure (%).

Healing			Mean		Percentiles			Range	
Period	Group	n	Mean	SD	Median	25	75	Min	Max
2 weeks	Sham	10	56.08 A	27.17	51.14	39.39	85.15	8.87	93.23
	DBBM + CM	10	26.30 B	16.73	32.09	8.53	39.80	0	48.1
	PLGA + β-TCP	9	37.48 AB	18.54	38.70	29.43	45.98	2.84	64.39
	PLGA + HA/β-TCP	10	49.56 AB	19.45	52.56	30.04	64.31	20.33	78.47
6 weeks	Sham	10	53.39 A	30.94	58.67	20.20	79.07	6.52	89.79
	DBBM + CM	10	57.50 A	28.57	51.11	32.10	82.47	25.38	100
	PLGA + β-TCP	10	52.77 A	26.91	52.04	35.70	83.54	9.42	84.52
	PLGA + HA/β-TCP	10	54.34 A	32.02	54.51	27.05	80.82	9.61	97.61

Estimates followed by different letters were statistically significant different (*p* < 0.05).

**Table 2:** Bone area fraction (%).

Healing			Mean		Percentiles			Range	
Period	Group	n	Mean	SD	Median	25	75	Min	Max
2 weeks	Sham	10	12.17 A	5.08	11.23	8.99	16.03	4.45	22.04
	DBBM + CM	10	6.32 B	8.72	3.46	2.16	6.74	0	30.21
	PLGA + β-TCP	9	4.71 B	2.96	5.36	2.88	6.69	0	8.91
	PLGA + HA/β-TCP	10	7.64 AB	5.60	6.69	2.97	11.37	1.93	17.51
6 weeks	Sham	10	17.84 A	10.30	18.31	8.64	26.90	3.01	33.01
	DBBM + CM	10	24.29 A	12.94	24.01	12.18	30.21	7.08	45.11
	PLGA + β-TCP	10	17.84 A	9.39	14.38	13.59	23.32	3.1	34.12
	PLGA + HA/β-TCP	10	18.11 A	12.78	14.80	7.30	27.15	4.88	41.66

Estimates followed by different letters were statistically significant different (*p* < 0.05).

**Table 3:** Histological biomaterial area fraction (%).

Healing			Mean		Percentiles			Range	
Period	Group	n	Mean	SD	Median	25	75	Min	Max
2 weeks	Sham	10	-	-	-	-	-	-	-
	DBBM + CM	10	32.96 A	14.45	39.96	18.08	42.71	5.56	50.41
	PLGA + β-TCP	9	40.82 A	21.63	48.24	32.82	49.64	5.17	77.69
	PLGA + HA/β-TCP	10	38.47 A	11.19	37.51	34.23	48.28	19.65	52.16
6 weeks	Sham	10	-	-	-	-	-	-	-
	DBBM + CM	10	31.04 A	10.88	35.15	18.89	38.67	12.18	41.86
	PLGA + β-TCP	10	34.05 A	13.15	31.71	22.88	46.32	15.20	52.10
	PLGA + HA/β-TCP	10	32.71 A	10.97	31.33	26.05	39.75	13.75	51.42

Estimates followed by different letters were statistically significant different (*p* < 0.05).

## Data Availability

Data may be available by request to the corresponding author.

## Abbreviations

The following abbreviations are used in this manuscript:

CM	collagen membrane
PLGA	poly-(lactic-co-glycolic-acid)
DBBM	deproteinized bovine bone mineral
HA	hydroxyapatite
β-TCP	β-tricalcium phosphate
μCT	microcomputed tomography
MTV	mineralized tissue volume
BIO	biomaterial volume
ICC	intraclass correlation

## Appendix A

**Table A1:** Controls and biomaterials.

Biomaterial	Composition	Particle Size (mm)	Source
Sham surgery	-	-	-
Bio-Oss	Bovine hydroxyapatite	0.25–1.00	Geistlich, Wolhusen, Switzerland
Easy-Graft Classic	Pure beta-tricalcium phosphate with poly(lactide-co-glycolide) + BioLinker (N-Methyl-2-pyrrolidone-solution)	0.5–1.00	Degradable Solutions AG, Schlieren, Switzerland
Easy-Graft Crystal	Biphasic calcium phosphate (60% hydroxyapatite, 40% beta-tricalcium phosphate) with poly(lactide-co-glycolide) + BioLinker (N-Methyl-2-pyrrolidone-solution)	0.45–1.00	Degradable Solutions AG, Schlieren, Switzerland
